# Ethnopharmacological-Based Validation of *Polyalthia suberosa* Leaf Extract in Neurological, Hyperalgesic, and Hyperactive Gut Disorders Using Animal Models

**DOI:** 10.1155/2022/1345006

**Published:** 2022-02-17

**Authors:** Ruhul Amin, Cristina Quispe, Jesús Herrera-Bravo, Md. Mizanur Rahman, Radmila Novakovic, Sevgi Durna Daştan, Atul Kabra, Javad Sharifi-Rad

**Affiliations:** ^1^Faculty of Pharmaceutical Science, Assam Down Town University, Panikhaiti, Guwahati, Assam, India; ^2^Facultad de Ciencias de La Salud, Universidad Arturo Prat, Avda. Arturo Prat 2120, Iquique 1110939, Chile; ^3^Departamento de Ciencias Básicas, Facultad de Ciencias, Universidad Santo Tomas, Chile; ^4^Center of Molecular Biology and Pharmacogenetics, Scientific and Technological Bioresource Nucleus, Universidad de La Frontera, Temuco 4811230, Chile; ^5^Pharmacy Department, Daffodil International University, Dhaka, Bangladesh; ^6^Institute of Pharmacology, Clinical Pharmacology and Toxicology, Medical Faculty, University of Belgrade, Belgrade, Serbia; ^7^Department of Biology, Faculty of Science, Sivas Cumhuriyet University, Sivas 58140, Turkey; ^8^Beekeeping Development Application and Research Center, Sivas Cumhuriyet University, Sivas 58140, Turkey; ^9^University School of Pharmaceutical Sciences, Chandigarh University, Gharuan, Mohali-140413, Punjab, India; ^10^Facultad de Medicina, Universidad del Azuay, Cuenca, Ecuador

## Abstract

*Polyalthia suberosa* (Roxb.) is a plant used to cure coughs, dysentery, fevers, joint aches, rheumatic pain, inflammation, and a variety of skin diseases. The aim of the study was to evaluate the ethyl acetate extract of *Polyalthia suberosa* (*P. suberosa*) leaves and their effects on mice for neuropharmacological, analgesic, and antidiarrheal activities. For neurological studies, the hole cross, hole board, open field, and thiopental sodium-induced sleep duration measurement methodologies were used. The castor oil-induced diarrhea inhibition test was used to assess antidiarrheal action, and the acetic acid-induced writhing inhibition test was used to determine analgesic effectiveness. The extract was given in doses of 250 and 500 mg kg^−1^ body weight. As a standard drug, diazepam at a dosage of 3 mg kg^−1^ was used. The extract was also given to groups, and sleep time was measured and recorded. The onset of the anxiolytic effect of the extract at both doses was found to be significant (*p* < 0.001), and sleep time increased to 273 minutes. For assessing analgesic activity, the extract along with standard diclofenac was administered and found to be 55.02 percent and 64.33 percent, respectively, for the extracts, and diclofenac was found to be 67.44 percent (*p* < 0.001). For antidiarrheal activity, it was compared with the standard drug, loperamide. The decrease for plant extracts was 50.07 percent and 70.06 percent at 250 mg kg^−1^ and 500 mg kg^−1^, respectively, whereas it was 85.01 percent for loperamide (3 mg kg^−1^) (*p* < 0.00). In this study, it was found that ethyl acetate extract of *Polyalthia suberosa* leaves had strong CNS depressant, analgesic, and antidiarrheal activities, which indicates that it may be used in contemporary medicine.

## 1. Introduction

Traditional medicine, like modern medicine, has exploded in popularity in Bangladesh in recent years. Traditional medicines (TM) in different forms have been utilized in our nation as a crucial way of treating illnesses and managing various health issues since the dawn of time [1]. In the previous several years, traditional remedies have seen exponential development in recent years, and these treatments are gaining appeal on both sides of the Atlantic. Because of their natural nature and fewer adverse effects, they are used in both developing and developed nations. There are several traditional medications available. Medicinal herbs, minerals, and organic substances are all used. The World Health Organization (WHO) has compiled a list of 21,000 plants utilized for medical reasons all around the globe [2, 3].

Sedatives and hypnotics are medicines that may relieve anxiety and provide a relaxing effect while allowing you to sleep. Currently, these medicines are widely used to treat a variety of mental problems, including anxiety and sleeplessness. Long-term use of these presently available sedative-hypnotic therapies, on the other hand, seems to have adverse effects ranging from respiratory, digestive, and immune system damage to decreased cognitive performance, physical dependence, and tolerance [4]. Numerous herbal medications have been shown to be active in the central nervous system (CNS) and may have the ability to treat chronic diseases including anxiety, depression, migraines, and epilepsy that do not respond well to conventional treatments. For many years, people in different parts of the globe have used herbal remedies to treat emotional disorders, and as a consequence, the search for new pharmacotherapy from medicinal plants has exploded in the past decade [5]. Medicinal plants are valuable treasures with a diverse range of compounds that are frequently used in conventional medicine to prevent, relieve, or cure a variety of human diseases in various parts of the world [6, 7], and about 80% of people in developing countries rely on herbal medicine to treat their illnesses [8]. There are a few medicinal plants that have neuropharmacological action and have fewer adverse effects than conventional medications. The presence of alkaloids, glycosides, and flavonoids in high concentrations in plant extracts has sedative, anxiolytic, and antiepileptic effects [9]. Analgesic effects are attributed to phenols and particular flavonoids such as normal, quercetin, and luteolin [10, 11]. Plants containing phytoconstituents such as tannins, alkaloids, saponins, flavonoids, steroids, and or terpenoids have antidiarrheal action [12]. *Polyalthia suberosa* (synonym: Uvaria suberosa Roxb.; family: Annonaceae) is a short, tiny tree that is extensively spread in Bangladesh, the West Indies, the Philippines, India, Sri Lanka, Malaysia, and Myanmar. Fruits are often used to prevent diarrhea. Lung issues are treated using fruits and herbs. Coughs, colds, and diarrhea are treated with the leaves. It is also utilized as an anti-HIV drug and for flatulence [13]. The bark is used as a febrifuge to prevent diarrhea and dysentery. This is an analgesic and laxative that is readily astringent. Seeds have a diuretic, sedative, and soporific action. Latex is utilized as an inexpensive filler for dental cavities in the tropics [14, 15]. The goal of this research was to see whether the ethyl acetate extract of *Polyalthia suberosa* leaves had any neuropharmacological, analgesic, or antidiarrheal properties.

## 2. Materials and Methods

### 2.1. Plant Collection and Extraction

During the month of December 2018, the leaves of *Polyalthia suberosa* were completely collected by us from Sundarban, Bangladesh. The sample was identified by Sr. Scientist Mahabuba Sultana at Bangladesh's National Herbarium in Mirpur, Dhaka. A voucher specimen (DACB: 46476) was submitted for future reference. The plant's leaves were gathered and cleaned in freshwater before being chopped into tiny pieces and shred-dried for up to ten days. Capacitor start motor, China dish, crushed the dried leaves into a fine powder. Cold extraction of 560 g of powder in 900 ml of 90% methanol in a clean and sealed glass jar for fourteen days at room temperature with periodic shaking and stirring yielded the crude extract. The whole combination was then coarsely filtered using a piece of clean, white cotton material, followed by filter paper. The extract was concentrated first in a rotary evaporator at low pressure and then in the open air. It was discovered that the yield was 5.5 percent w/w. In all the studies, freshly produced extracts were utilized.

### 2.2. Phytochemical Screening

Using conventional chemical assays, several phytochemical groups such as alkaloids, glycosides, flavonoids, tannins, gums, saponins, and steroids have been recognized by distinctive color change. The presence of carbohydrates was determined using the Mulish and Fehling tests [16, 17]. Proteins were detected using the Biuret test. The presence of phytochemical analysis in plant extracts and ethanolic aqueous solutions was determined using the following conventional procedures [18–21].

#### 2.2.1. Test for Tannins

The 0.5 g extract was mixed with 10 ml of bromine water. The presence of tannins was shown by the decolorization of bromine water.

#### 2.2.2. Test for Saponins

In a test tube, 5.0 mL of distilled water was combined with an aqueous crude plant extract and aggressively stirred. The foam was combined with a few drops of olive oil and violently agitated, revealing the presence of saponins in the foam.

#### 2.2.3. Tests for Flavonoids

*(1) Shinoda Test*. Magnesium ribbons and concentrated HCl were combined with aqueous crude plant extract, and the pink hue indicated flavonoid content.

*(2) Alkaline Reagent Test*. We combined 2 ml of 2.0 percent NaOH with aqueous plant crude extract and obtained an intense yellow tint, which became colorless when we added 2 drops of diluted acid to the mixture. Flavonoids were found in this sample.

#### 2.2.4. Tests for Glycosides

*(1) Liebermann's Test*. We mixed aqueous plant crude extract with 2.0 mL of acetic acid and 2 mL of chloroform. After cooling the liquid, we added concentrated H_2_SO_4_. The entity of an aglycone, the steroidal portion of glycosides, was shown in green.

*(2) Keller–Kiliani Test*. A 4.0 mL solution of glacial acetic acid was combined with 10 mL of aqueous plant extract and 1 mL of concentrated H_2_SO_4_ to make a Keller–Kiliani combination. Between the layers, a brown ring developed, indicating the presence of cardiac steroidal glycosides.

*(3) Salkowski's Test*. To the aqueous plant crude extract, we added 2 mL of concentrated H_2_SO_4_. The presence of the steroidal aglycone portion of the glycoside resulted in a reddish-brown tint.

#### 2.2.5. Test for Terpenoids

The 5 ml of aqueous plant extract was mixed with 2.0 ml of chloroform and evaporated on the water route before being heated with 3 ml of concentrated H_2_SO_4_. The entity of terpenoids appeared as a grayish color.

#### 2.2.6. Test for Steroids

To the 5 ml of aqueous plant crude extract, 2 ml of chloroform and concentrated H_2_SO_4_ were added. A red hue occurred in the bottom chloroform layer, indicating the presence of steroids.

### 2.3. Experimental Animals

This study utilized Swiss albino mice that were 4-5 weeks old and weighed 25–28 g on average. The mice were procured from the Pharmacology Lab, Pharmacy Department of Jahangirnagar University. The mice were maintained in a regular setting for 10 days and were given a standard pellet diet and free access to water. The study protocol was authorized by Daffodil International University's Institutional Animal Ethics Committee in Dhaka, Bangladesh (Ref. FAHSREC/DIU/2019/1005, dated December 6, 2019).

### 2.4. Evaluation of Neuropharmacological Activity

#### 2.4.1. Thiopental Sodium-Induced Sleeping Time

The test was carried out according to the instructions [22]. Negative control groups, positive control groups, and test groups were used to split the mice into four groups (I and II). In four cages, each group of six mice was maintained separately. The mice in the negative control group were given 10 ml kg^−1^ body weight of 1 percent (v/v) Tween-80 orally in distilled water. The mice in the positive control group were given 3 mg kg^−1^ body weight of the conventional medication diazepam. Mice in research groups I and II were given crude extract at dosages of 250 and 500 mg kg^−1^ body weight, respectively. With the use of a needle, all dosages were given orally. After 30 minutes, all sleep-inducing groups received thiopental sodium (40 mg kg ^−1^) intraperitoneally. All the animals began to sleep at the same time. The mice were placed in the inverted posture after being put to sleep, and after the sedation was finished, the mice returned to normal, and the time was recorded. The hypnotic effect index was calculated as the time between the loss and recovery of the righting reflex. Latency was defined as the period between the thiopental sodium injection and the onset of sleep. The following formula was used to determine the percentage of effect:

(1) $$\begin{array}{c} \text{effect}\left(\text{\%}\right)=\frac{\text{average duration of loss of righting reflex in the test group}}{\text{average duration of loss of righting reflex in the control group}}\times 100. \end{array}$$

#### 2.4.2. Open Field Test

In this study, 24 mice were chosen at random and split into four groups: negative control, positive control, and test groups (I and II). The negative control group received just 1% (*v*/*v*) Tween-80 at a dosage of 10 ml kg^−1^ body weight, whereas the positive group received the standard medication, diazepam, as an oral solution at a dose of 3 mg kg^−1^ body weight. The research groups (I and II) were given oral doses of 250 mg kg^−1^ and 500 mg kg^−1^ body weight of plant extract solution. Orally, both dosages were given with the help of sterile feeding. The animals were placed separately in one of the corners of square grids (100 cm × 100 cm × 40 cm) after being diagnosed. The number of squares traversed by the mice was tracked for 3 minutes at 0, 30, 60, 90, and 120 minutes throughout the observation period. The experiment was conducted in a completely quiet setting [23].

#### 2.4.3. Hole Board Test

The mice were split into four groups, each with six mice weighing 25–28 g. Group I received 1 percent Tween-80, Group II received diazepam at a dosage of 3 mg kg^−1^ body weight, and Groups III and IV received 250 mg kg^−1^ and 500 mg kg^−1^ body weight, respectively, of ethyl acetate extract of *Polyalthia suberosa* (leaves). Mice were placed near the screen's edge at the start of the experiment. For the whole observation period, the number of head dips in the holes for a period of 3 minutes was tallied at 0, 30, 60, 90, and 120 minutes. The experiment was carried out in a sound-proofed room [24].

#### 2.4.4. Hole Cross Test

Following their respective care of the community, the mice were separately placed in the cage's darker chamber, divided by a wall with a hole in the dark and white chambers. At 0, 30, 60, 90, and 120 minutes, the total number of crossings from one chamber to the other by the mice of each group was tallied. The experiment was carried out in a sound-proofed room [25, 26].

### 2.5. Evaluation of Analgesic Activity

#### 2.5.1. Acetic Acid-Induced Writhing Test

For the analgesic test, the mice were split into four groups. Each group consists of six mice. The first group is for distilled water and is a controlled group. Group 2 was the control group, which received diclofenac sodium BP (10 mg kg^−1^). Alcoholic extract (250 mg kg^−1^ and 500 mg kg^−1^) was given to groups 3 and 4. After 45 minutes, each mouse was given a 10 ml kg^−1^ body weight injection of 0.7 percent acetic acid. After 15 minutes of IP administration of acetic acid, the number of writhing responses for each animal was recorded for 3 minutes, and the mean abdominal writhes for each group were calculated [27]. The percentage inhibition was calculated using the following formula:

(2) $$\begin{array}{c} \text{inhibition }\left(\text{\%}\right)=\frac{\text{mean number of writhes }\left(\text{control}\right)-\text{mean number of writhes }\left(\text{drugs}\right)}{\text{mean number of writhes }\left(\text{control}\right)}. \end{array}$$

### 2.6. Evaluation of Antidiarrheal Activity

#### 2.6.1. Castor Oil-Induced Diarrheal Test

All animals were checked for diarrhea before the experiment by giving them 0.5 ml of castor oil orally, and the animals that started having diarrhea were chosen for the test. Following the screening, twenty-six mice were chosen and split into four groups, each with six mice. Prior to the trial, mice were fasted for 18 hours with free access to water. Group 1 received distilled water (10 ml kg^−1^ body weight) as a control, whereas Group 2 received a standard treatment (loperamide 3 mg kg^−1^ body weight). The extract was administered in different dosages to groups 3 and 4 (250 mg kg^−1^ and 500 mg kg^−1^, respectively). Both mice were given 0.5 mL of castor oil orally 30 minutes after the treatment to initiate diarrhea and were placed in cages on blotting paper. The paper may be changed at any time. For each group of animals, the quantity of diarrheal feces was recorded and the percentage of defecation inhibition was computed throughout the 4 h observation period [28, 29].

### 2.7. Data Analysis

The results are presented as an average SEM. One-way variance analysis (ANOVA) was used in the statistical analysis, which was followed by Dunnett's post hoc test, a sleep time test, and a hole board test. Two-way ANOVA was used for the hole cross test and open field test, followed by Bonferroni's post hoc testing. The statistical analysis was carried out with the help of Microsoft Excel 2007 software.

## 3. Results

### 3.1. Phytochemical Screening

The presence of reducing tannin, alkaloid, glycoside, steroid, flavonoid, phenol, carbohydrate, and terpenoid, as well as the lack of saponins, was found in the crude ethyl acetate extract of *Polyalthia suberosa* (leaves) in a chemical community (Table 1).

### 3.2. Evaluation of Neuropharmacological Activity

#### 3.2.1. Thiopental Sodium-Induced Sleeping Time

When we gave *Polyalthia suberosa* leaf extract at 250 mg kg^−1^ and 500 mg kg^−1^ dosages to thiopental sodium patients, their sleeping duration was altered. In comparison with the control group, the behavior of extracts shows that it lowers latent duration and boosts or prolongs the sleeping time (Table 2).

#### 3.2.2. Open Field Test

Although the impact of diazepam was greater compared to the results of crude extracts, the crude extracts exhibited a statistically significant decrease in mouse movements when compared to placebo. At each measured dosage of 250, 500, and 1000 mg kg^−1^, decreased activity was seen at the second observation and lasted until the fourth observation (Table 3).

#### 3.2.3. Hole Cross Test

**Figure 1:**
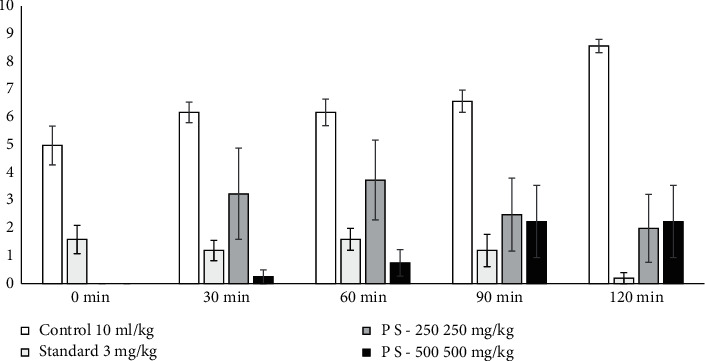
Polyalthia suberosa on the hole cross test in mice.

Although the impact of diazepam was strong compared to the crude extract testing, the crude extracts demonstrated a statistically significant decrease in locomotor activity in mice at any dosage tested (250 and 500 mg kg^−1^). The 2nd persistent observation to the 4th observation revealed a decrease in locomotor operation (Table 4 and Figure 1).

#### 3.2.4. Hole Board Test

**Figure 2:**
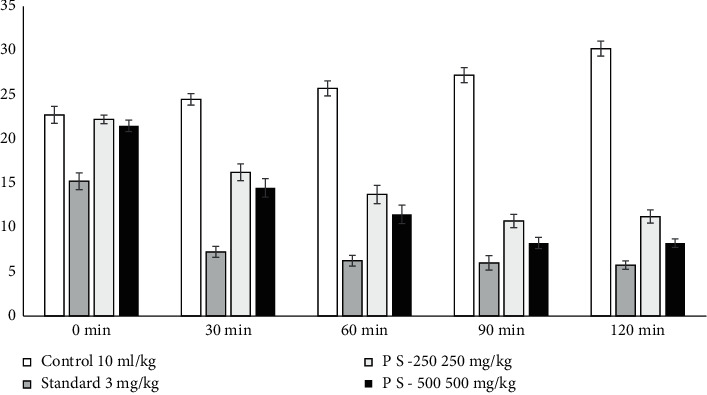
*Polyalthia suberosa* on the hole board test in mice.

Although the impact of diazepam was strong compared to the findings of the crude extracts, the crude extracts at each dosage exhibited a significant decrease in the frequency of head dips relative to the placebo in the hole board check. The effect started on the experiment's second observation and continued until the fourth observation (Table 5 and Figure 2).

### 3.3. Evaluation of Analgesic Activity

#### 3.3.1. Acetic Acid-Induced Writhing Test

At oral dosages of 250 and 500 mg kg^−1^ body weights of mice, the crude extract inhibited writhing by 55.02 percent and 64.33 percent, respectively. The conventional medication diclofenac sodium, on the other hand, showed a 67.44 percent inhibition at a dose of 10 mg kg^−1^ body weight (Table 6).

### 3.4. Evaluation of Antidiarrheal Activity

#### 3.4.1. Castor Oil-Induced Test

**Figure 3:**
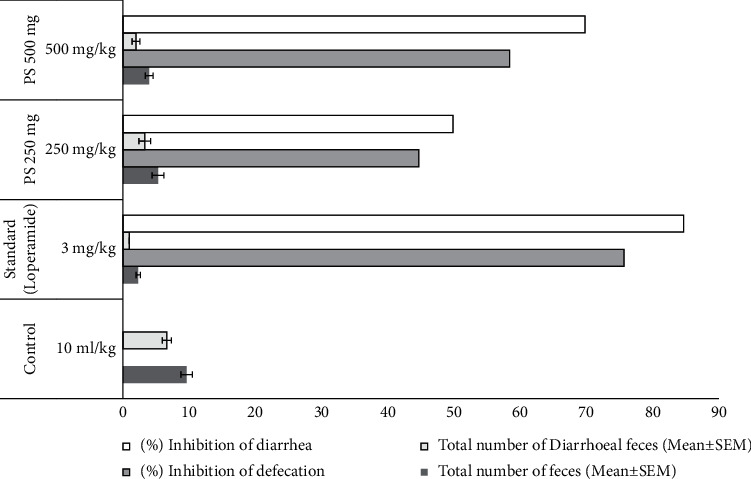
Neuropharmacological, analgesic, and antidiarrheal activity of *Polyalthia suberosa* leaf extract in Swiss albino mice.

The extract had a significant antidiarrheal effect in mice in a castor oil-induced diarrhea test. When compared to the untreated control group, the extract significantly reduced the incidence of defecation (*p* 0.001) and dose lowered the total quantity of diarrheal feces. At 250 and 500 mg kg^−1^ dosages of the extract, the percentage inhibition of diarrhea was 50.07 percent and 70.06 percent, respectively. The percentage suppression of diarrhea by loperamide HCl (3 mg kg^−1^) was 85.01 percent (*p* < 0.001, vs control) (Table 7 and Figure 3).

## 4. Discussion

Phytochemical analysis revealed the presence of alkaloids, tannins, terpenoids, flavonoids, phenols, and steroids in *Polyalthia suberosa* extracts in the current study. The existence of these phytochemical substances has been linked to *Polyalthia suberosa*'s biological activity. The effects of secondary bioactive metabolites of *Polyalthia suberosa* on neuropharmacology, analgesia, and antidiarrhea were investigated in this study. Vivo hypnosis, open field, hole board, and hole cross methods all provide neuropharmacological impact. Beyond basic movement, the open field test assesses a variety of aspects of behavior [30, 31].

The open board test (HBT) is a scientific research method for measuring anxiety, tension, and emotion in animals [32]. It is possible to measure a wide range of processes because of its measurement capabilities. It is a common test in pharmacology, but the results are not always obvious. External symptoms were estimated through hole cross checks to complete the approval of anxiousness. Some studies have found that plant extracts rich in alkaloids, glycosides, and flavonoids have sedative, anxiolytic, and antiepileptic properties that interfere with their properties at the benzodiazepine site of the GABAergic complex structure or are immediate or aberrant modulators that are responsible for increases in GABA activity in the brain that cause drowsiness and facies [33, 34]. It seems that the phytochemicals included in the extract of *Polyalthia suberosa* leaves contribute to the relaxing and sleep-inducing actions on the CNS, at least to a limited degree. We started our investigation to see how the effect of *Polyalthia suberosa*'s CNS wretchedness differs from the uncontrolled locomotory behavior of mice in hole cross and open field experiments. The oral organization of the test removal at dosages of 250 and 500 mg kg^−1^ resulted in a substantial decrease in the number of holes traversed and sleepiness to a new condition, which was reversed when the CNS stimulating agent was used. Each of the parts that were tested got a motion critique. Another big impact was created, and this one was much worse than the hole board test. By examining the exploratory behaviors of rats, this test is established as a method of evaluating the potential anxiolytic and narcotic effects of any operator. The behavior of animals plummeting their heads is definitely linked to their mental tension [35]. According to this view, the declaration of anxiolytic condition in animals may be represented by an increase in head-plunging behavior [36], while a decrease in head-plunging was regarded as related to the depressive effect [37, 38].

The acetic acid-induced writhing procedure was used to evaluate the extract's analgesic effectiveness. Acetic acid raised the levels of local endogenous chemicals such as PGE2, PGF2, lipoxygenase products, acetylcholine, and histamine, which are responsible for pain sensation in the peritoneal fluids [39–41]. As the concentration of the extract was raised, the inhibition of writhing in mice grew, and the former acquired a remarkable inhibition of writhing similar to normal diclofenac sodium (Table 6). Analgesic action is known to be mediated by a variety of flavonoids, alkaloids, and steroids [42]. The phytochemical community of leaf extract research has revealed several important phytochemicals, such as alkaloids, tannins, and flavonoids. These polyphenolic chemicals are thought to be responsible for the leaf extract's analgesic action.

Diarrhea is described as irregular defecation with a poor consistency of feces, which may be caused by a disturbance in water and electrolyte transport in the intestines. The active metabolite of castor oil, ricinoleic acid, stimulates the release of endogenous prostaglandins and peristaltic activity in the small intestine, resulting in alterations in the intestinal mucosa's electrolyte permeability [43, 44]. The crude ethyl acetate extract of *Polyalthia suberosa* (leaves) showed a statistically significant decrease in the incidence and frequency of diarrhea in experimental animals, according to our findings. In the castor oil-induced diarrhea research in mice, the *Polyalthia suberosa* extract exhibited a significant antidiarrheal effect at both dosages.

## 5. Conclusion

The results of this research show that *Polyalthia suberosa* leaf extract possesses outstanding antidepressant, analgesic, and antidiarrheal properties. In mice, the extract depresses the CNS and possesses numerous sedative and anticonvulsant properties. Finally, the results of this study provide evidence for the plant's potential application in ethnomedicine while also providing early data on the plant's core behavior. Consequently, these findings offer fresh information on plant development, which may lead to the identification of medicines derived from natural sources. To isolate the bioactive chemicals and explain the precise processes responsible for the pharmacological actions found in this plant, further chemical and pharmacological research is needed.

## Figures and Tables

**Table 1:** Phytochemical test results of extract of *Pterocarpus indicus*.

Tested groups	Ethyl acetate extract of *P. suberosa* (leaves)
Tannins	+
Alkaloids	+
Glycosides	+
Saponins	−
Steroids	+
Flavonoids	+
Phenol	+
Carbohydrate	+
Terpenoid	+

**Table 2:** Effect of *Polyalthia suberosa* on thiopental sodium-induced sleeping time in mice (mean ± SEM, *n* = 6).

Group	Dose	Latent period	Sleeping time	% effect
Control (1% v/v Tween-80: water)	10 ml kg^−1^	87.33 ± 1.45	136.67 ± 9.53	0.0
Standard (diazepam)	3 mg kg^−1^	2.6 ± 0.88	337.8 ± 14.19^*∗∗∗*^	96.94
PS-250	250 mg kg^−1^	40 ± 7.21^*∗∗∗*^	251.67 ± 6.57^*∗∗∗*^	51.53
PS-500	500 mg kg^−1^	50.66 ± 3.84^*∗∗∗*^	273 ± 8.76^*∗∗∗*^	41.01

^*∗*^indicates *p* < 0.05,  ^*∗∗*^indicates *p* < 0.01, and  ^*∗∗∗*^indicates *p* < 0.001 when compared with control.

**Table 3:** Effect of *Polyalthia suberosa* on the open field test in mice (mean ± SEM, *n* = 6).

Group	Dose	Number of movements (% of movement inhibition)				
		0 min	30 min	60 min	90 min	120 min
Control	10 ml kg^−1^	26.5 ± 0.64	27.75 ± 0.47	28.0 ± 0.63	27.5 ± 0.65	27.5 ± 0.64
Standard	3 mg kg^−1^	20.5 ± 0.28^*∗∗∗*^	13.0 ± 0.41^*∗∗∗*^	10.5 ± 0.65^*∗∗∗*^	9.25 ± 0.48^*∗∗∗*^	8.25 ± 0.48^*∗∗∗*^
PS-250	250 mg kg^−1^	24.0 ± 0.40^*∗∗*^	18.25 ± 0.47^*∗∗∗*^	16.5 ± 0.64^*∗∗∗*^	15.55 ± 0.64^*∗∗∗*^	15.73 ± 0.85^*∗∗∗*^
PS-500	500 mg kg^−1^	23.25 ± 0.48^*∗∗*^	16.0 ± 0.41^*∗∗∗*^	13.5 ± 0.65^*∗∗∗*^	13.25 ± 0.48^*∗∗∗*^	13.5 ± 0.64^*∗∗∗*^

^*∗*^indicates *p* < 0.05,  ^*∗∗*^indicates *p* < 0.01, and  ^*∗∗∗*^indicates *p* < 0.001 when compared with control.

**Table 4:** Effect of *Polyalthia suberosa* on the hole cross test in mice (mean ± SEM, *n* = 6).

Group	Dose	Number of head dips				
		0 min	30 min	60 min	90 min	120 min
Control	10 ml kg^−1^	5 ± 0.70	6.2 ± 0.37	6.2 ± 0.48	6.6 ± 0.40	8.6 ± 0.24
Standard	3 mg kg^−1^	1.6 ± 0.51	1.2 ± 0.37^*∗*^	1.6 ± 0.40^*∗∗*^	1.2 ± 0.58^*∗∗*^	0.2 ± 0.20^*∗∗*^
PS-250	250 mg kg^−1^	0.0 ± 0.0	3.25 ± 1.65	3.75 ± 1.44^*∗*^	2.5 ± 1.32	2.0 ± 1.23^*∗∗*^
PS-500	500 mg kg^−1^	0.0 ± 0.0	0.25 ± 0.25^*∗*^	0.75 ± 0.48^*∗∗*^	2.25 ± 1.31	2.25 ± 1.31^*∗∗*^

^*∗*^indicates *p* < 0.05,  ^*∗∗*^ indicates *p* < 0.01, and  ^*∗∗∗*^ indicates *p* < 0.001 when compared with control.

**Table 5:** Effect of *Polyalthia suberosa* on the hole board test in mice (mean ± SEM, *n* = 6).

Group	Dose	Number of movements (% of movement inhibition)				
		0 min	30 min	60 min	90 min	120 min
Control	10 ml kg^−1^	22.75 ± 0.95	24.5 ± 0.65	25.75 ± 0.85	27.25 ± 0.85	30.25 ± 0.85
Standard	3 mg kg^−1^	15.25 ± 0.95*∗∗∗*	7.25 ± 0.63^*∗∗∗*^	6.25 ± 0.62^*∗∗∗*^	6 ± 0.82^*∗∗∗*^	5.75 ± 0.48^*∗∗∗*^
PS-250	250 mg kg^−1^	22.25 ± 0.48	16.25 ± 0.95^*∗∗∗*^	13.75 ± 1.03^*∗∗∗*^	10.75 ± 0.75^*∗∗∗*^	11.25 ± 0.75^*∗∗∗*^
PS-500	500 mg kg^−1^	21.5 ± 0.65	14.5 ± 1.05^*∗∗∗*^	11.5 ± 1.04^*∗∗∗*^	8.25 ± 0.63^*∗∗∗*^	8.25 ± 0.48^*∗∗∗*^

^*∗*^indicates *p* < 0.05,  ^*∗∗*^indicates *p* < 0.01, and  ^*∗∗∗*^indicates *p* < 0.001 when compared with control.

**Table 6:** Analgesic activity of *Polyalthia suberosa* on acetic acid-induced writhing in mice (mean ± SEM, *n* = 6).

Treatment	Dose	Number of writhing (mean ± SEM)	% inhibition
Control (distilled water)	10 ml kg^−1^	21.50 ± 2.92	00
Standard (diclofenac sodium)	10 mg kg^−1^	7.0 ± 0.52^*∗∗∗*^	67.44%
PS-250	250 mg kg^−1^	9.67 ± 1.38^*∗∗∗*^	55.02%
PS-500	500 mg kg^−1^	7.67 ± 0.72^*∗∗∗*^	64.33%

^*∗*^indicates *p* < 0.05,  ^*∗∗*^indicates *p* < 0.01, and  ^*∗∗∗*^indicates *p* *<* 0.001 when compared with control.

**Table 7:** Effect of *Polyalthia suberosa* in the castor oil-induced diarrheal test.

Group	Dose	Total number of feces (mean ± SEM)	% inhibition of defecation	Total number of diarrhoeal feces (mean ± SEM)	% inhibition of diarrhea
Control	10 ml kg^−1^	9.67 ± 0.88	00	6.67 ± 0.67	00
Standard (loperamide)	3 mg kg^−1^	2.33 ± 0.33	76	1 ± 0.0^*∗∗∗*^	85.01
PS-250 mg	250 mg kg^−1^	5.33 ± 0.88	44.89	3.33 ± 0.89^*∗∗∗*^	50.07
PS-500 mg	500 mg kg^−1^	4 ± 0.58	58.64	2 ± 0.58^*∗∗∗*^	70.06

^*∗*^indicates *p* < 0.05,  ^*∗∗*^indicates *p* < 0.01, and  ^*∗∗∗*^indicates *p* < 0.001 when compared with control.

## Data Availability

The data used to support the findings of this study are available from the corresponding author upon request.
